# Comparison of short-term outcomes of combined neoadjuvant chemotherapy and immunotherapy, neoadjuvant radiotherapy, and neoadjuvant chemotherapy for resectable locally advanced esophageal squamous cell carcinoma

**DOI:** 10.3389/fmed.2025.1682377

**Published:** 2025-12-08

**Authors:** Hai Zhang, Zicheng Dai, Bei Wang, Bomeng Wu, Jiangbo Lin, Wanli Lin

**Affiliations:** 1 Department of Thoracic Surgery, Afiliated Gaozhou People’s Hospital, Guangdong Medical University, Guangdong, China; 2 Department of Thoracic Surgery, Fujian Medical University Union Hospital, Fuzhou, China

**Keywords:** esophageal squamous cell carcinoma, neoadjuvant therapy, immunotherapy, chemoradiotherapy, short-term efficacy

## Abstract

**Background:**

This study aimed to evaluate the safety and efficacy of neoadjuvant immunochemotherapy (nICT) compared to those of neoadjuvant chemoradiotherapy (nCRT) and neoadjuvant chemotherapy (nCT) in patients with locally advanced resectable esophageal squamous cell carcinoma (ESCC).

**Methods:**

Patients with locally advanced resectable ESCC undergoing neoadjuvant therapy followed by minimally invasive McKeown esophagectomy (MIE) between January 1, 2019, and January 1, 2022, were categorized into the nCT, nCRT, and nICT groups. Inverse probability of treatment weighting (IPTW) was used to balance the 13 baseline covariates across the groups. Post-IPTW comparisons included adverse events, surgical outcomes, pathologic complete response (PCR), tumor downstaging, and perioperative complications.

**Results:**

A total of 437 patients were enrolled (nICT = 218, nCRT = 78, nCT = 141). After propensity score matching (PSM), 78 patients were included in each group. During neoadjuvant therapy, the incidence of leukopenia was significantly higher in the nCRT (33.33%) and nICT (23.08%) groups than in the nCT (10.26%), *p* < 0.05. Neutropenia rates followed a similar trend (nCRT: 19.23%; nICT: 17.95%; nCT: 2.56%, *p* < 0.05). Immune-related dermatitis occurred in 10.26% of patients with nICT. Operative duration was significantly longer with nCRT (347.8 ± 80.8 min) than nCT (325.1 ± 52.6 min; *p* < 0.05). The tumor-downstaging efficacy was ranked as follows: nICT > nCRT > nCT (*p* < 0.05). Lymph node dissection:Yields: nCRT (26 ± 11) < nICT (36 ± 14) and nCT (35 ± 12) (*p* < 0.05),Stations: nICT (13 ± 4) > nCRT (9 ± 2) and nCT (11 ± 2) (*p* < 0.05). The PCR rates were 34.61% (nCRT), 26.92% (nICT), and 5.13% (nCT), respectively (*p* < 0.05). Postoperative 30-day mortality occurred in three nCRT and one nCT patients. Postoperative arrhythmia incidence was highest with nICT (12.82%), intermediate with nCT (5.13%), and absent with nCRT (0.00%).

**Conclusion:**

For locally advanced resectable ESCC, nCRT requires vigilant management because of its association with grade 3 treatment-related adverse events (AEs). While nICT demonstrates a lower incidence of severe (grade ≥ 3) non-immune toxicities compared to nCRT, supporting its controllable safety. nCRT achieved superior PCR rates versus nICT/nCT, but nICT showed greater nodal (N) downstaging efficacy. Conversely, nCRT provided enhanced primary tumor (T) control.

## 1 Introduction

Esophageal squamous cell carcinoma (ESCC) is a highly prevalent malignant gastrointestinal tumor in China, with locally advanced resectable tumors occupying a significant proportion (1). Although radical surgery is the mainstay of treatment, the recurrence rate after surgery alone is high and long-term survival is unsatisfactory. Neoadjuvant therapy has become an important strategy to improve the prognosis of such patients. Based on the CROSS NEOCRTEC5010 study, neoadjuvant radiotherapy has been recommended as a standard treatment by several guidelines, which can significantly increase the R0 resection rate, rate of pathological complete remission, and improve survival (2, 3). However, the toxic side effects of nCRT and its poor efficacy in some patients remain clinical challenges. Neoadjuvant chemotherapy was shown to be efficacious in some studies, particularly in specific populations (4). In recent years, immune checkpoint inhibitors have aided the treatment of advanced esophageal cancer, significantly prolonging patient survival (5–7). This has prompted investigators to explore neoadjuvant immunotherapy. Neoadjuvant chemotherapy combined with immunotherapy as an emerging strategy, has been investigated in a series of preliminary clinical trials in resectable esophageal cancer, exhibiting appreciable rates of pathological remission, including superiority over nCT, similarity to nCRT, and safe, compliant rates of adverse events (8). The effects of immunotherapy could theoretically be augmented by the tumor antigen-releasing effects of chemotherapy, promising a new and potentially effective alternative to nCRT with different toxicity profiles.

However, there is a lack of sufficient comparative evidence on the short-term efficacy (e.g., pathologic remission rate, R0 resection rate, and treatment-related adverse events) of the three neoadjuvant treatment strategies (nICT, nCRT, and nCT) in patients with resectable locally advanced ESCC. Short-term efficacy metrics (e.g., PCR rate) have been shown to be crucial surrogate endpoints for predicting long-term survival benefit. Therefore, a systematic comparative assessment of the short-term efficacy and safety of these three regimens is clinically relevant for optimizing neoadjuvant treatment choices for locally advanced ESCC and guiding individualized precision treatment decisions. This study aimed to evaluate the differences in short-term efficacy and safety among nCIT, nCRT, and nCT in patients with resectable locally advanced ESCC through a retrospective comparative analysis.

## 2 Materials and methods

### 2.1 Study design

This study used a retrospective analysis to enroll patients with locally advanced resectable esophageal squamous cell carcinoma who were treated with neoadjuvant therapy combined with minimally invasive McKeown’s surgery at the Gaosu City People’s Hospital and the Union Hospital of Fujian Medical University from 2019-01-01 to 2022-01-01 and were divided into nCT, nCRT, and nICT groups according to neoadjuvant therapy protocols.

The inclusion criteria for patient selection were listed as:

1. Age 18–75 years, both sexes; ECOG score–0–1; 2. Pretreatment clinically diagnosed thoracic esophageal squamous cell carcinoma, with the treatment plan comprising of combined neoadjuvant therapy and minimally invasive McKeown surgery; 3. No evidence of chronic diseases or chronic comorbidities, and 4. Normal sensorium and vital status.

The exclusion criteria for this study were: 1. Patients with a history of severe psychological disorders 2. Patients receiving hormone replacement therapy, 3. Clinical evidence of malignant tumors other than esophageal cancer; and 4. Chronic comorbidities or diseases that might affect patient’s short term survival.

### 2.2 Observational indicators

Clinical data were collected primarily from an inpatient electronic case system, outpatient visit records, and archived medical records. Follow-up data were obtained mainly through telephonic follow-up, WeChat, other software communications, and medical records in the hospital.

The data collected mainly included (1) patients’ demographic data before treatment, (2) patient’s baseline characteristics such as height, weight, Body Mass Index (BMI), weight loss in 3 months prior to treatment, history of comorbidities, and patient’s personal data, (3) tumor-related indices before treatment: esophageal tumor location and clinical stage of the tumor, according to the eighth edition of the American Joint Committee on Cancer/National Comprehensive Cancer Network (AJCC/NCCN) guidelines, (4) data obtained during the neoadjuvant therapy cycle: records of the occurrence of adverse events during neoadjuvant therapy, and the grade of adverse events using the Common Terminology Criteria for Adverse Events (CTCAE) version 5.0^1^ grading method.

Additionally, information on perioperative clinical data and postoperative pathological tumor regression grading (using American Pathological Association standards) were also obtained (9).

### 2.3 Treatment regimen

The nCT regimen: TP regimen, PF regimen, and NP regimen were as follows: (1) use of paclitaxel chemotherapeutic drugs (paclitaxel injection 135–175 mg/m^2^ or docetaxel injection 75 mg/m^2^ or paclitaxel albumin-bound 260 mg/m^2^); (2) Fluorouracil (750 mg–1,000 mg/m^2^) d1–d4); (3) Vincristine (25 mg/m^2^ d1,8,15,22); combined with platinum-based chemotherapy drugs (cisplatin 60–100 mg/m^2^ or carboplatin 0.3–0.4 g/m^2^ or nedaplatin 80–100 mg/m^2^), q3w, chemotherapy drugs used for 2–4 cycles, according to the chemotherapy drugs and the patient’s response to the appropriate dose adjustment.

The nCRT regimen comprised of: radiotherapy using an appropriate intensity-modulated radiotherapy technique: 20 times, GTV: 40–54 Gy/20–24 F, CTV: 40–44 Gy/20 F, during which paclitaxel-based chemotherapeutic drugs (paclitaxel injection 135–175 mg/m^2^ or docetaxel injection 75 mg/m^2^ or paclitaxel albumin-bound 260 mg/m^2^) or fluorouracil (750–1,000 mg−1,000 mg/m^2^) were administered. Additionally, 750 mg–1,000 mg/m^2^ d1–d4) or vincristine (25 mg/m^2^ d1,8,15,22) combined with platinum-based chemotherapeutic agents (cisplatin 60–100 mg/m^2^ or carboplatin 0.3–0.4 g/m^2^ or nedaplatin 80–100 mg/m^2^), chemotherapeutic agents were used for 2∼4 cycles, and the dosage were adjusted according to chemotherapeutic agents and patients’ responses.

nICT regimen: The chemotherapy regimen (q3w, 2–4 cycles) was the same as the neoadjuvant chemotherapy. Immunotherapeutic drugs were administered before chemotherapy (navulizumab 3 mg/kg, d1, q3w; pembrolizumab 2 mg/kg, d1, q3w; karelizumab 200 mgd1, q3w; sindilizumab, 200 mgd1, q3w; and tirilizumab, 200 mgd1, q3w).

Surgical treatment plan: A minimally invasive McKeown surgical plan was used, and a two-field sweep was routinely performed to clear the right mediastinum and epigastric region lymph nodes, whereas a three-field sweep (sweeping the neck, mediastinum, and epigastric region draining lymph nodes) was chosen for patients with preoperative evaluation of the possible presence of cervical lymph node metastasis.

### 2.4 Statistical analysis

All statistical analyses were performed using SPSS19.0 (IBM Corporation, Armonk, NY, United States). Age, BMI, operative time, intraoperative bleeding, number of lymph node dissection, number of lymph node dissection stations, chest tube drainage time, and postoperative hospitalization days were expressed as mean ± standard deviation using Student’s *t*-test. Sex, pre-treatment comorbidities, history of smoking and alcohol consumption, hemoglobinopenia, leukopenia, neutropenia, thrombocytopenia were recorded in both groups. Pearson’s chi-squared test was computed for the rate of elevation of alanine aminotransferase/glutamyl aminotransferase, total bilirubin, creatinine levels, vomiting, diarrhea, incidence of postoperative pulmonary infections, postoperative pathological vasculature invasion, and postoperative pathological neurological invasion. The pre-treatment tumor location was assessed using the likelihood ratio test. Clinical T and N staging and cTNM staging, postoperative pathological staging, and postoperative pathological tumor regression (TRG) grading were performed using the Mann—Whitney U test. Postoperative cardiac arrhythmia, derangements in liver and renal functions, complications of pleural effusion, anastomotic fistula, thoracogastric fistula, bronchial fistula, secondary surgery, celiac chest, voice hoarseness rate, postoperative dysphagia rate, postoperative blood transfusion, 30-day mortality rate, and 30-day unplanned admission rate were analyzed using the Fisher’s exact test.

The baseline characteristics of the three patient groups was analyzed using Propensity Score Matching (PSM). Propensity scores were calculated using Multinomial Logistic Regression (MLR) and adjusted using Inverse Probability of Treatment Weighting (IPTW) to weigh the covariate distributions of the three groups of patients and converge to the overall distribution. Data analysis was performed in the RStudio software environment using the R language package “pm3” (version number: 10.32614/CRAN.Package.pm3) for propensity score matching.

Graphs used in this study were created using the CNSknowall program.^2^

## 3 Results

### 3.1 Baseline clinical characteristics

A total of 437 patients were included in the study. According to the different neoadjuvant treatment protocols, the patients were divided into neoadjuvant chemotherapy (*n* = 141), neoadjuvant chemoradiotherapy (*n* = 78), and chemotherapy combined with immunotherapy (*n* = 218) groups. The baseline data of the patients in the three groups included demographic characteristics, vital statistics, and pretreatment oncological characteristics (Table 1). Smoking history (definition 0: no smoking, 1: smoking index 1–400 years of cigarettes, 2: smoking index 400–800 years of cigarettes, and 3: smoking index greater than 800 years of cigarettes).

**TABLE 1:** Comparison of baseline data among the three groups of patients.

Variable	nCRT (*n* = 78)	nICT (*n* = 218)	nCT (*n* = 141)	*p*-value	SMD
Male, n (%)	57(73.1)	171 (78.4)	109(77.3)	0.625	0.083
Age (years), mean (SD)	61.37(7.10)	60.52 (6.60)	61.16 (7.38)	0.549	0.081
BMI (kg/m^2^), mean (SD)	21.93(2.73)	22.29(12.71)	22.23 (2.72)	0.957	0.052
Weight loss (+), n (%)	5 (6.4)	22 (10.1)	18 (12.8)	0.330	0.145
Hypertension (+), n (%)	6 (7.7)	36 (16.5)	31 (22.0)	0.025	0.273
CHD (+), n (%)	1 (1.3)	1 (0.5)	5 (3.5)	0.073*	0.152
Diabetes (+), n (%)	4 (5.1)	15 (6.9)	9 (6.4)	0.863	0.049
Smoking, n (%)				0.098*	0.140
0	47 (60.3)	99 (45.4)	64 (45.4)		
1	2 (2.6)	19 (8.7)	13 (9.2)		
2	10 (12.8)	39 (17.9)	33 (23.4)		
3	19 (24.4)	61 (28.0)	31 (22.0)		
Alcohol (+), n (%)	18 (23.1)	63 (28.9)	43 (30.5)	0.492	0.112
Tumor location, n (%)				0.008	0.266
Upper	7 (9.0)	18 (8.3)	12 (8.5)		
Middle	56 (71.8)	111 (50.9)	70 (49.6)		
Lower	15 (19.2)	89 (40.8)	59 (41.8)		
cTNM, n (%)				0.967	0.052
II	28 (35.9)	80 (36.7)	56 (39.7)		
III	45 (57.7)	123 (56.4)	77 (54.6)		
IV	5 (6.4)	15 (6.9)	8 (5.7)		
cT, n (%)				0.377*	0.176
2	9 (11.5)	45 (20.6)	32 (22.7)		
3	66 (84.6)	165 (75.7)	104 (73.8)		
4	3 (3.8)	8 (3.7)	5 (3.5)		
cN, n (%)				0.725*	0.106
0	22 (28.2)	42 (19.3)	30 (21.3)		
1	45 (57.7)	145 (66.5)	90 (63.8)		
2	9 (11.5)	21 (9.6)	15 (10.6)		
3	2 (2.6)	10 (4.6)	6 (4.3)		

CHD, Coronary heart disease; SMD, Standardized mean difference; SD, Standard deviation. Adjusted *p*-values marked with asterisk “*.”

The baseline data of the three groups of patients showed differences in pre-treatment combined with hypertension and tumor lesion location, *p* < 0.05. To reduce selection bias and make the pretreatment baseline data of the three groups of patients consistent, the distribution of covariates among the three groups was balanced by propensity score matching (PSM), which enhanced the comparability between the groups (Table 2). (10).

**TABLE 2:** Comparison of baseline data among the three groups after PSM.

Variable	nCRT (*n* = 78)	nICT (*n* = 78)	nCT (*n* = 78)	*p*-value	SMD
Male, n (%)	57(73.1)	63(80.8)	60(76.9)	0.522	0.121
Age (years), mean (SD)	61.37 (7.10)	62.17 (6.58)	61.35 (7.63)	0.716	0.078
BMI (kg/m^2^), mean (SD)	21.93 (2.73)	22.13 (2.35)	22.10 (2.75)	0.873	0.051
Weight loss (+), n (%)	5 (6.4)	5 (6.4)	19(12.8)	0.255	0.145
Hypertension (+), n (%)	6 (7.7)	8 (10.3)	14 (17.9)	0.121	0.206
CHD (+), n (%)	1 (1.3)	0 (0)	4 (5.1)	0.070*	0.235
Diabetes (+), n (%)	4 (5.1)	5 (6.4)	5 (6.4)	0.927*	0.036
Smoking, n (%)				0.065*	0.036
0	47 (60.3)	29 (37.2)	36 (46.2)		
1	2 (2.6)	6 (7.7)	7 (9.0)		
2	10 (12.8)	15 (19.2)	17 (21.8)		
3	19 (24.4)	28 (35.9)	18 (23.1)		
Alcohol (+), n (%)	18 (23.1)	20 (25.6)	22 (28.2)	0.764	0.270
Tumor location, n (%)				0.085	0.078
Upper	7 (9.0)	7 (9.0)	8 (10.3)		
Middle	56 (71.8)	41 (52.6)	43 (55.1)		
Lower	15 (19.2)	30 (38.5)	27 (34.6)		
cTNM, n (%)				0.558	0.163
II	28 (35.9)	19 (24.4)	27 (34.6)		
III	45 (57.7)	52 (66.7)	45 (57.7)		
IV	5 (6.4)	7 (9.0)	6 (7.7)		
cT, n (%)				0.719*	0.100
2	9 (11.5)	12 (15.4)	15 (19.2)		
3	66 (84.6)	63 (80.8)	59 (75.6)		
4	3 (3.8)	3 (3.8)	4 (5.1)		
cN, n (%)				0.653*	0.199
0	22 (28.2)	13 (16.7)	16 (20.5)		
1	45 (57.7)	49 (62.8)	47 (60.3)		
2	9 (11.5)	11 (14.1)	11 (14.1)		
3	2 (2.6)	5 (6.4)	4 (5.1)		

CHD, Coronary heart disease; SMD, Standardized mean difference. Adjusted *p*-values marked with asterisk “*.”

### 3.2 Occurrence of relevant adverse events during the neoadjuvant therapy cycle

During the neoadjuvant therapy cycle, eight cases of immune-related rashes of different grades were observed in the nICT group, of which one case had a grade 4 reaction that required readmission to the hospital and discontinuation of the second course of neoadjuvant therapy. Immune-related dermatitis occurred in 10.26% of patients with nICT.

Two cases of radiation pneumonitis and one case of esophageal fistula occurred in the nCRT group. Meanwhile, there were seven cases of unplanned discontinuation in the nCRT group due to toxic side effects that were not tolerated by the patients, or the patients refused the next course of treatment.

To understand the variability in the occurrence of adverse events during neoadjuvant therapy among the different neoadjuvant regimens, a two-by-two comparative approach was used (Tables 3–5).

**TABLE 3:** Comparison of the incidence of adverse events during neoadjuvant therapy cycles between nCRT and nICT.

Variable	nCRT (*n* = 78)	nICT (*n* = 78)	χ^2^/Z	*p*-value
Anemia, n (%)			3.830	0.280
Grade 1	18(23.08)	26(33.33)		
Grade 2	5(6.41)	3(3.85)		
Grade 3	0(0.00)	1(1.28)		
Leukopenia, n (%)			6.919	0.009
Grade 1	9(11.54)	14(17.95)		
Grade 2	11(14.10)	4(5.13)		
Grade 3	6(7.69)	0(0.00)		
Neutropenia, n (%)			0.018	0.893
Grade 1	11(14.10)	11(14.10)		
Grade 2	3(3.85)	1(1.28)		
Grade 3	1(1.28)	2(2.56)		
Thrombocytopenia, n (%)			4.707	0.095
Grade 1	4(5.13)	1(1.28)		
Grade 2	0(0.00)	2(2.56)		
Elevated bilirubin, n (%)			1.393	0.498
Grade 1	1(1.28)	1(1.28)		
Grade 2	0(0.00)	1(1.28)		
Elevated ALT, n (%)			2.126	0.345
Grade 1	2(2.56)	4(5.13)		
Grade 2	0(0.00)	1(1.28)		
Elevated AST, n (%)	1(1.28)	2(2.56)		1.000
Elevated creatinine, n (%)	2(2.56)	0(0.00)		0.497
Vomiting (≥ Grade 2), n (%)	6(7.69)	3(3.85)	0.472	0.492

**TABLE 4:** Comparison of adverse event rates within the neoadjuvant therapy cycle of nCRT versus nCT.

Variable	nCRT (*n* = 78)	nCT (*n* = 78)	χ^2^/Z	*p*-value
Anemia, n (%)			0.356	0.551
Grade 1	18(23.08)	24(30.77)		
Grade 2	5(6.41)	0(0.00)		
Grade 3	0(0.00)	0(0.00)		
Leukopenia, n (%)			16.485	0.001
Grade 1	9(11.54)	7(8.97)		
Grade 2	11(14.10)	1(1.28)		
Grade 3	6(7.69)	0(0.00)		
Neutropenia, n (%)			10.419	0.001
Grade 1	11(14.10)	2(2.56)		
Grade 2	3(3.85)	0(0.00)		
Grade 3	1(1.28)	0(0.00)		
Thrombocytopenia, n (%)	4(5.13)	0(0.00)	2.309	0.129
Elevated bilirubin, n (%)	1(1.28)	0(0.00)		1.000
Elevated ALT, n (%)	2(2.56)	1(1.28)	0.000	1.000
Elevated AST, n (%)	1(1.28)	0(0.00)		1.000
Elevated creatinine, n (%)	2(2.56)	0(0.00)		0.487
Vomiting (≥ Grade 2), n (%)	6(7.69)	3(3.85)	0.472	0.492

**TABLE 5:** Comparison of adverse event rates within neoadjuvant therapy cycles for nICT versus nCT.

Variable	nICT(n = 78)	nCT(n = 78)	χ^2^/Z	*p*-value
Anemia, n (%)			5.978	0.113
Grade 1	26(33.33)	24(30.77)		
Grade 2	3(3.85)	0(0.00)		
Grade 3	1(1.28)	0(0.00)		
Leukopenia, n (%)			4.820	0.028
Grade 1	14(17.95)	7(8.97)		
Grade 2	4(5.13)	1(1.28)		
Neutropenia, n (%)			12.048	0.007
Grade 1	11(14.10)	2(2.56)		
Grade 2	1(1.28)	0(0.00)		
Grade 3	2(2.56)	0(0.00)		
Thrombocytopenia, n (%)			4.299	0.117
Grade 1	1(1.28)	0(0.00)		
Grade 2	2(2.56)	0(0.00)		
Hyperbilirubinemia, n (%)			2.799	0.247
Grade 1	1(1.28)	0(0.00)		
Grade 2	1(1.28)	0(0.00)		
Elevated ALT, n (%)			3.420	0.181
Grade 1	4(5.13)	1(1.28)		
Grade 2	1(1.28)	0(0.00)		
Elevated AST, n (%)	2(2.56)	0(0.00)		0.497

### 3.3 Comparison of perioperative related indexes among three groups of patients after neoadjuvant therapy

#### 3.3.1 Comparison of indicators related to surgical techniques

Among the three groups of patients, surgery was transferred to an open chest/open abdomen. One patient in the nCRT group was considered to have a tumor involving the right lower pulmonary vein, which could not be resected by R0 under the microscope, and was then transferred to open thoracic surgery for treatment.

Among the three groups of patients, according to the surgical record documentation and postoperative pathological confirmation, R0 surgical resection was not performed in two patients, one case each in the nCRT group and the nCT group, which were preoperative patients with cT4a, and after neoadjuvant therapy, the descending stage was not obvious, which led to the inability of R0 resection, and the postoperative pathological TRG grading suggested a grade 3 response.

**FIGURE 1:**
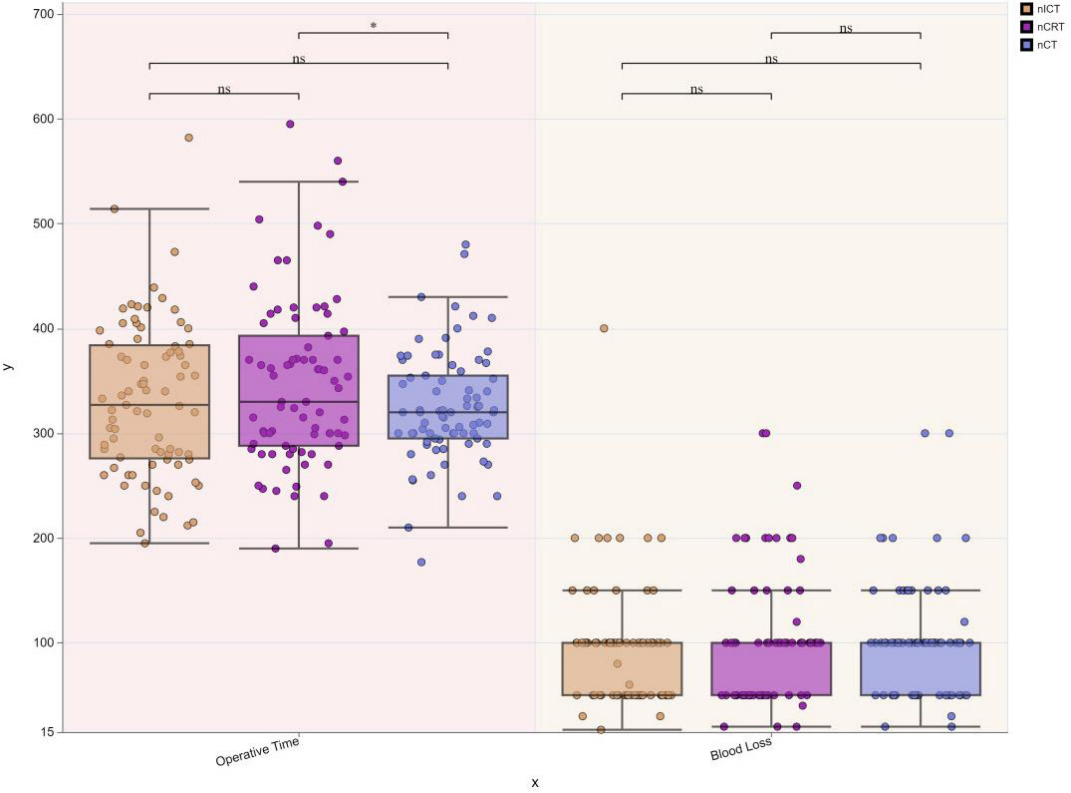
Comparative analysis of operative metrics. The study incorporated comparisons of intraoperative blood loss and operative time. Operative time:nCRT group: 347.794 ± 80.837 min, nICT group: 330.820 ± 74.026 min, nCT group: 325.064 ± 52.567 min. Intraoperative blood loss:nCRT group: 104.487 ± 62.991 mL, nICT group: 93.807 ± 55.326 mL, nCT group: 101.794 ± 53.494 mL. Difference boxplots were used to compare the variability of these two metrics across the three groups. The boxplots revealed that the nCRT group had the longest operative time, while the nCT group had the shortest. No statistically significant difference was observed in operative time between the nICT group and either the nCRT or nCT groups (*p* > 0.05). However, the operative time in the nCRT group was significantly longer than that in the nCT group (*p* < 0.05). Regarding intraoperative blood loss, no statistically significant differences were found among the three groups (*p* > 0.05). * indicates *p* < 0.05; ns (no significance) indicates *p* > 0.05.

Comparisons of intraoperative bleeding and operative time were included in the study, and different box plots were used to compare the three groups of patients for these two indicators (Figure 1).

#### 3.3.2 Postoperative pathology reporting of the three groups of patients

**FIGURE 2:**
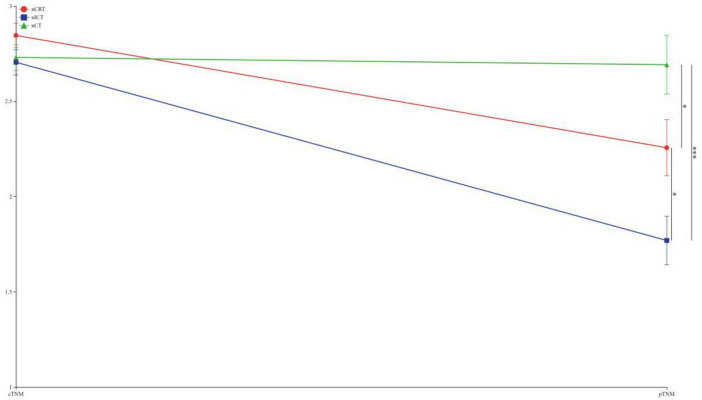
Line chart of TNM staging differences before and after treatment. *p < 0.05; ***p < 0.005.

**FIGURE 3:**
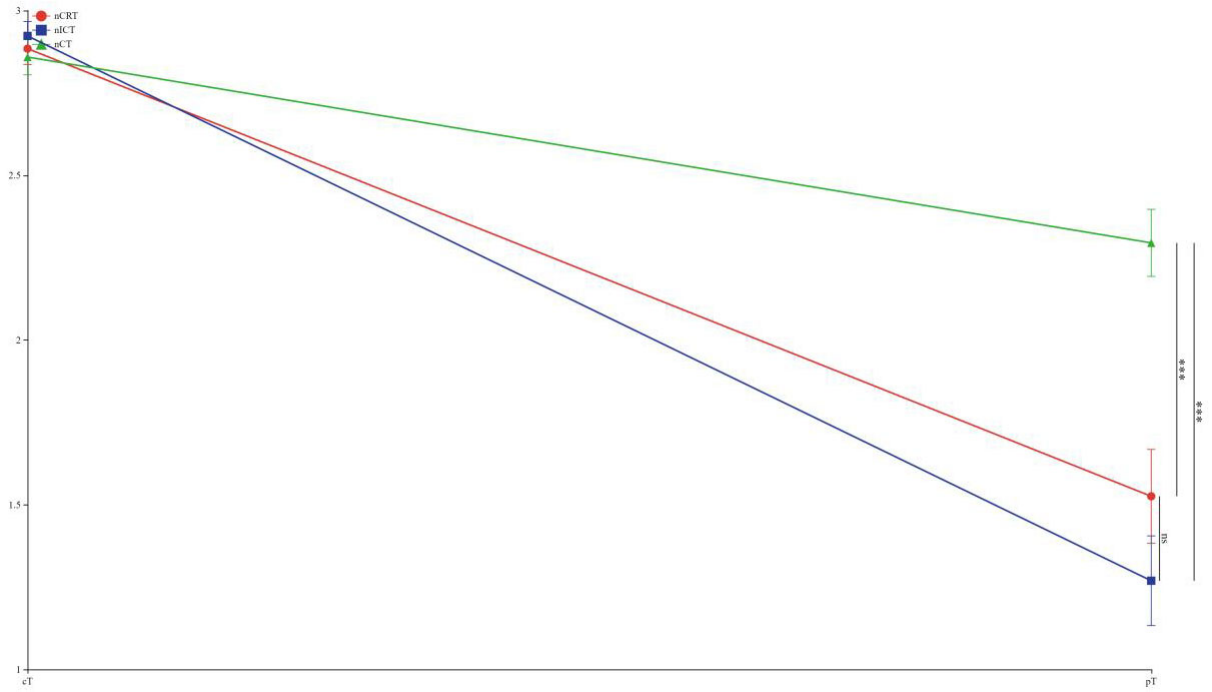
Line chart of T staging differences before and after treatment. ***p < 0.005; ns, not significant.

**FIGURE 4:**
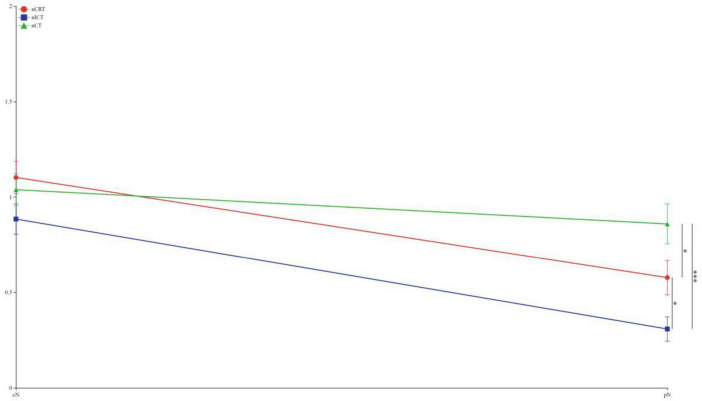
Line chart of n staging differences before and after treatment. *p < 0.05; **p < 0.01; ***p < 0.005; ns, not significant.

**FIGURE 5:**
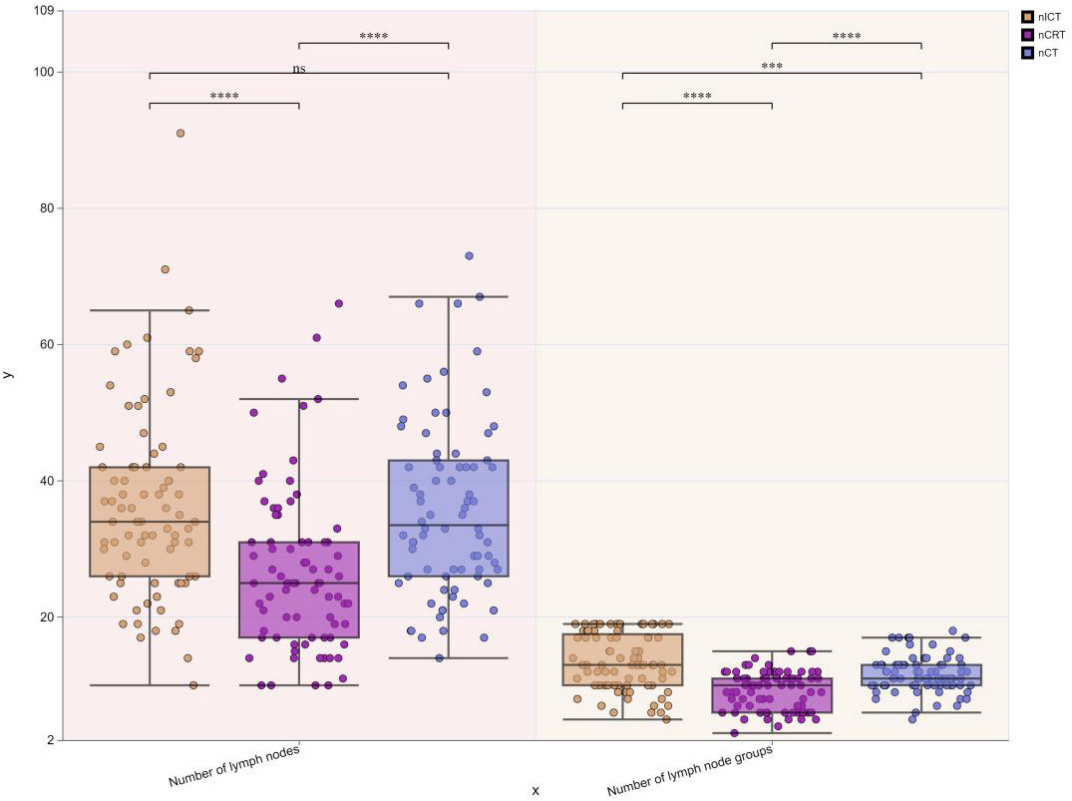
Boxplots of differences in lymph node retrieval count and dissected stations among three treatment groups. *p < 0.05; **p < 0.01; ***p < 0.005; ****p < 0.001; ns, not significant.

To visually express the trends and differences between the cTNM staging before neoadjuvant therapy and the corresponding ypTNM staging of the three groups of patients, the following line graphs of the differences in the three modes of treatment are depicted nCRT treatment demonstrates a more pronounced rightward shift, indicating a superior downstaging pattern, followed by nICT. In contrast, the connecting lines between pre-treatment cTNM and post-treatment pTNM in nCT remain relatively balanced, suggesting limited downstaging efficacy. Furthermore, a statistically significant difference (*p* < 0.05) is observed between nCRT and nICT. Compared with nCT, nICT exhibits significantly better downstaging effects (*p* < 0.05) (Figure 2). The line chart comparing pre- and post-treatment T staging reveals no statistically significant difference (ns, *p* > 0.05) in T downstaging efficacy between nCRT and nICT. However, both nCRT and nICT demonstrate significantly superior T downstaging results compared to nCT, with a statistically significant difference (*p* < 0.005) (Figure 3). Comparison of N staging differences before and after treatment reveals that nICT demonstrates significantly superior N downstaging results compared to nCRT (*p* < 0.05). Additionally, nCRT also shows better efficacy in N downstaging than nCT, with a statistically significant difference (*p* < 0.05) (Figure 4). Lymph node retrieval count:nCRT group: 26 ± 11 nodes, nICT group: 36 ± 14 nodes, nCT group: 35 ± 12 nodes. Number of dissected lymph node stations:nCRT group: 9 ± 2 stations, nICT group: 13 ± 4 stations, nCT group: 11 ± 2 stations. The nCRT group demonstrated significantly fewer lymph nodes retrieved and dissected stations compared to both nICT and nCT groups (*p* < 0.05). While no significant difference existed between nICT and nCT groups in lymph node retrieval count (ns, *p* > 0.05), the nICT group had significantly more dissected stations than the nCT group (*p* < 0.05) (Figure 5).

**FIGURE 6:**
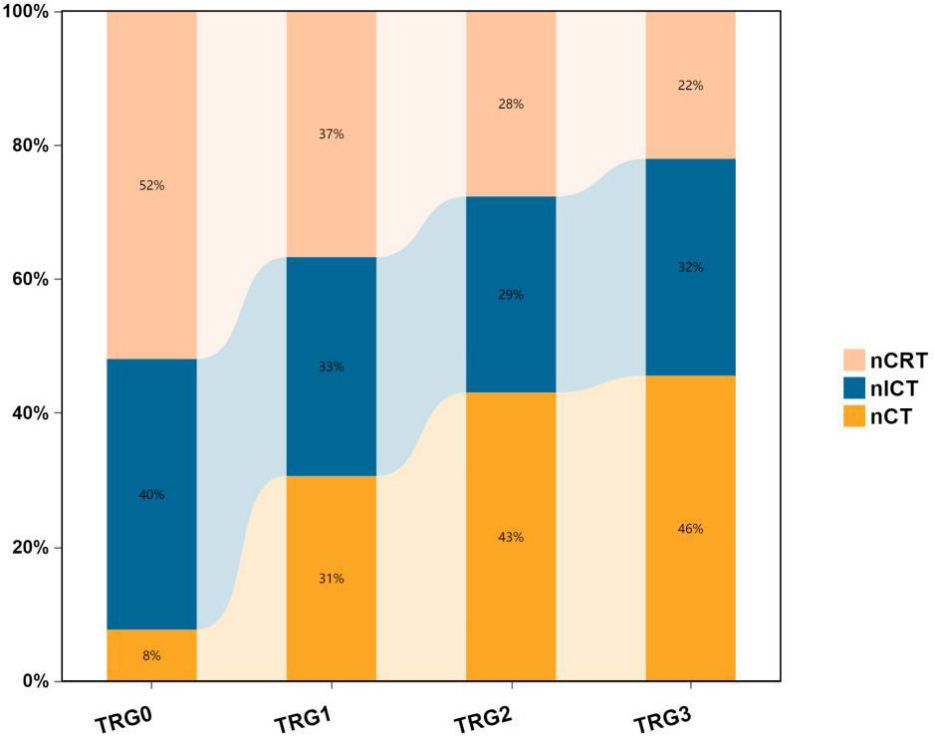
Stacked percentage bar chart of pathological TRG grades among three treatment groups.

The chart demonstrates that the nCRT group exhibits a higher proportion of TRG 0 compared to nICT and nCT groups, indicating superior pathological regression and higher pCR (pathological complete response) rates, while the nCT group shows relatively lower pCR rates. Furthermore, the stacked bar chart reveals distinct distribution patterns:nCRT group: Displays a favorable downward trend in TRG grades, with the highest proportions in TRG 0 and TRG 1.nICT group: Demonstrates relatively balanced distribution across TRG grades, with a moderately higher proportion in TRG 0.nCT group: Exhibits an unfavorable upward trend, dominated by TRG 2 and TRG 3 responses (Figure 6).

TRG grading, the presence of vascular invasion, and nerve invasion were compared among the three groups of patients (Table 6).

**TABLE 6:** Comparison of postoperative pathologic TRG grading, vascular invasion, and nerve invasion among the three groups of patients.

Variable	nCRT(*n* = 78)	nICT(*n* = 78)	nCT(*n* = 78)	X^2^	*p*-value
TRG grade, n (%)				21.380	0.001
0	27(34.61)	21(26.92)	4(5.13)		
1	18(23.07)	16(20.51)	15(19.23)		
2	18(23.07)	19(24.26)	28(35.90)		
3	15(19.23)	22(28.21)	31(39.74)		
Lymphovascular invasion, n (%)	9(11.54)	7(8.97)	12(15.38)	1.535	0.464
Perineural invasion, n (%)	8(10.26)	12(15.38)	21(26.92)	7.832	0.020

#### 3.3.3 Postoperative care-related data of patients in the three groups

**FIGURE 7:**
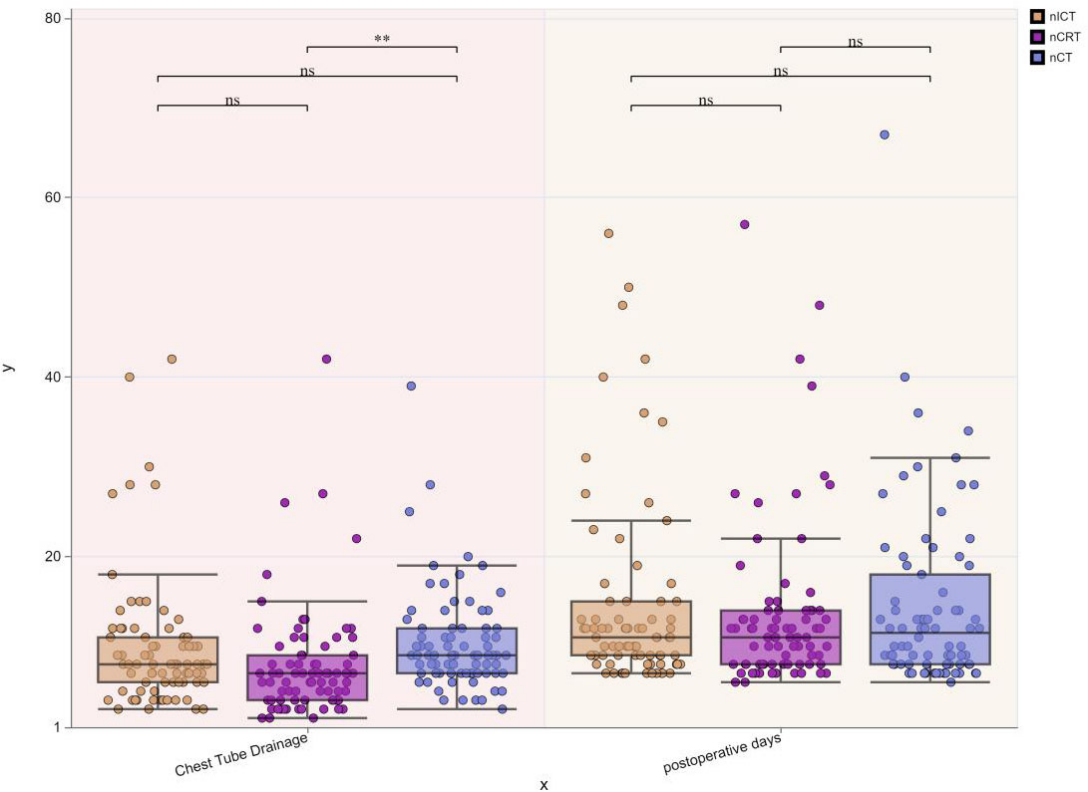
Boxplots of postoperative chest tube duration and hospital stay among three treatment groups. **p < 0.01; ns, not significant.

Postoperative chest tube duration:nCRT group: 7.86 ± 6.21 days, nICT group: 9.95 ± 7.51 days, nCT group: 10.44 ± 5.66 days. The nCT group showed significantly longer chest tube duration compared to the nCRT group (*p* < 0.05). Postoperative hospital stay:nCRT group: 13.74 ± 9.40 days, nICT group: 14.55 ± 10.67 days, nCT group: 14.42 ± 9.92 days. No statistically significant differences (ns, *p* > 0.05) were observed in postoperative hospital stay among the three groups (Figure 7).

#### 3.3.4 Comparison of postoperative complications among the three groups of patients

The details of the postoperative complications in the three patient groups are shown in Tables 7–9.

**TABLE 7:** Comparison of nCRT and nICT groups in terms of postoperative complications.

Variable	nCRT (*n* = 78)	nICT (*n* = 78)	χ^2^/Z	*p*-value
Postoperative pneumonia, n (%)	22(28.21)	22(28.21)	0.000	1.000
Postoperative arrhythmia, n (%)	0(0.00)	10(12.82)	8.655	0.003
Postoperative liver dysfunction, n (%)	6(7.69)	12(15.38)	2.261	0.133
Postoperative pleural effusion, n (%)	1(1.28)	10(12.82)	7.922	0.005
Postoperative renal dysfunction, n (%)	0(0.00)	1(1.28)	1.393	0.236
Anastomotic leakage, n (%)	14(17.95)	8(10.26)	1.905	0.168
Gastric conduit fistula, n (%)	3(3.85)	2(2.56)	0.208	0.648
Bronchial fistula, n (%)	2(2.56)	0(0.00)	2.799	0.094
Reoperation, n (%)	0(0.00)	1(1.28)	1.393	0.236
Chylothorax, n (%)	1(1.28)	1(1.28)	0.000	1.000
Postoperative blood transfusion, n (%)	1(1.28)	3(3.85)	1.073	0.300
30-day mortality, n (%)	3(3.85)	0(0.00)	4.218	0.040
30-day readmission, n (%)	2(2.56)	2(2.56)	0.000	1.000
ICU readmission, n (%)	2(2.56)	6(7.69)	1.186	0.276
Hoarseness, n (%)	8(10.26)	8(10.26)	0.000	1.000
Dysphagia, n (%)	4(5.13)	0(0.00)	5.650	0.017

**TABLE 8:** Comparison of nCRT and nCT groups in terms of postoperative complications.

Variable	nCRT (*n* = 78)	nCT (*n* = 78)	χ^2^/Z	*p*-value
Postoperative pneumonia, n (%)	22(28.21)	24(30.77)	0.123	0.725
Postoperative arrhythmia, n (%)	0(0.00)	4(5.13)	5.650	0.017
Postoperative liver dysfunction, n (%)	6(7.69)	24(30.77)	13.371	0.001
Postoperative pleural effusion, n (%)	1(1.28)	16(20.51)	14.854	0.001
Postoperative renal dysfunction, n (%)	0(0.00)	3(3.85)	4.218	0.040
Anastomotic leakage, n (%)	14(17.95)	9(11.54)	1.275	0.259
Gastric conduit fistula, n (%)	3(3.85)	3(3.85)	0.000	1.000
Bronchial fistula, n (%)	2(2.56)	1(1.28)	0.346	0.556
Reoperation, n (%)	0(0.00)	1(1.28)	1.393	0.236
Chylothorax, n (%)	1(1.28)	1(1.28)	0.000	1.000
Postoperative blood transfusion, n (%)	1(1.28)	0(0.00)	1.393	0.236
30-day mortality, n (%)	3(3.85)	1(1.28)	1.073	0.300
30-day readmission, n (%)	2(2.56)	0(0.00)	2.799	0.094
ICU readmission, n (%)	2(2.56)	7(8.97)	3.112	0.078
Hoarseness, n (%)	8(10.26)	7(8.97)	0.074	0.786
Dysphagia, n (%)	4(5.13)	4(5.13)	0.000	1.000

**TABLE 9:** Comparison of postoperative complications between the nICT and nCT groups.

Variable	nCT (*n* = 78)	nICT (*n* = 78)	χ^2^/Z	*p*-value
Postoperative pneumonia, n (%)	24(30.77)	22(28.21)	0.123	0.725
Postoperative arrhythmia, n (%)	4(5.13)	10(12.82)	2.825	0.093
Postoperative liver dysfunction, n (%)	24(30.77)	12(15.38)	5.200	0.023
Postoperative pleural effusion, n (%)	16(20.51)	10(12.82)	1.662	0.197
Postoperative renal dysfunction, n (%)	3(3.85)	1(1.28)	1.073	0.300
Anastomotic leakage, n (%)	9(15.38)	8(10.26)	0.066	0.797
Gastric conduit fistula, n (%)	3(3.85)	2(2.56)	0.208	0.648
Bronchial fistula, n (%)	1(1.28)	0(0.00)	1.393	0.236
Reoperation, n (%)	1(1.28)	1(1.28)	0.000	1.000
Chylothorax, n (%)	1(1.28)	1(1.28)	0.000	1.000
Postoperative blood transfusion, n (%)	0(0.00)	3(3.85)	4.218	0.040
30-day mortality, n (%)	1(1.28)	0(0.00)	1.393	0.236
30-day readmission, n (%)	0(0.00)	2(2.56)	2.799	0.094
ICU readmission, n (%)	7(8.97)	6(7.69)	0.084	0.772
Hoarseness, n (%)s	7(8.97)	8(10.26)	0.074	0.786
Dysphagia, n (%)	4(5.13)	0(0.00)	5.650	0.017

## 4 Discussion

### 4.1 Comparison of adverse events associated with three treatment regimens during neoadjuvant therapy

The rate of adverse events during the neoadjuvant therapy cycle is the primary reference index for assessing the safety of neoadjuvant therapy. Adverse events during the neoadjuvant treatment cycle were compared among the three neoadjuvant treatment modalities to further explore the safer and clinically actionable treatment regimen, including healthcare team operability and patient compliance. During treatment, the percentage of patients who failed to follow the course of treatment in the nCRT group was 8.97% owing to related toxic side effects, while there was only one case of grade 4 rash in the nICT group, which led to discontinuation of the second course of neoadjuvant therapy. This shows the preference of nCRT clinical implementation in patients, leading to an out-of-trial risk.

After propensity score matching, there were 78 patients in each group. The overall occurrence of grade III adverse events was 4.27%, out of which 70% were centered on myelosuppressive reactions in the nCRT group, whereas 20% myelosuppression occurred in the nICT group. In this study, the total percentage of leukopenia cases at all levels was 33.33% in the nCRT group, which was a higher incidence relative to both the nCT and nICT groups. In a neoadjuvant therapy study based on the CROSS protocol, a 22% incidence of grade 4 neutropenia was reported (11). Similarly, leukopenia and neutropenia were reported as major complications of nCRT in NEOCRTEC5010, with incidences of 48.9 and 45.7%, respectively (12). In the comparison between the nICT and nCT groups, it was found that the incidence of myelosuppression was higher in the nICT group than in the nCT group. In this study, nCT did not exhibit definite grade III myelosuppressive reactions but showed a statistically significant difference based on data from the current study, which showed that the incidence of myelosuppression in nICT was in the range of 20% (13). This requires clinical intervention, although the incidence of myelosuppression with nICT treatment, especially grade 3 or higher, is low compared to nCRT, and its incidence is high compared to nCT.

Esophageal perforation is a serious adverse event during neoadjuvant therapy and can occur either as a result of damage from treatment or necrosis of the tumor itself due to the treatment response. The exact incidence of esophageal perforation during or after neoadjuvant therapy could not be accurately confirmed. However, based on previous reports, high-risk factors for esophageal perforation include nCRT and cT4, and perforation is associated with radiotherapy regimens and individual patient differences (14, 15). Although Van Hagen et al. reported a low incidence of esophageal perforation during nCRT (1/171), this complication severely impacts treatment continuity and is associated with high mortality during neoadjuvant therapy. Esophageal perforation has been recognized as one of the most critical and potentially fatal adverse events in patients undergoing neoadjuvant therapy (8). In this study, one case of esophageal perforation occurred during nCRT and was treated with left thoracic surgery; this was also one of the cases that caused perioperative death.

In this study, the myelosuppressive response assessment combined multidimensional indicators (leukopenia, neutropenia, and thrombocytopenia). Overall, the incidence of grade 3 or higher myelosuppressive adverse events during nCRT was higher than that during nICT, which in turn was higher than that during nCT. However, a meta-analysis by Wang et al. showed that nICT may increase the incidence of adverse events compared to chemotherapy alone (16). However, in a meta-analysis that included 27 clinical studies with 815 patients, Ge et al. reported a combined incidence of treatment-related serious adverse events of 26.9% (95% CI, 16.7∼38.3%), and the overall incidence of grade 3 or higher adverse events did not progress further compared to nCT (17). This discrepancy may be related to the chemotherapeutic regimen and immunologic agents used. Based on studies on nIT for esophageal cancer, the addition of ICIs during neoadjuvant therapy may increase the incidence of immune-related adverse events; however, the incidence of immunotherapy-related adverse events, according to previous reports, is inconsistent. In addition, the specific type and severity of adverse events may vary depending on the study design, immunological agent, and combination chemotherapy regimen (18, 19). During nCRT treatment, attention should be paid to the possibility of esophageal perforation, and patients should be adequately evaluated for imaging data, radiotherapy planning, and alternative regimens before treatment. During the course of nICT treatment, the occurrence of immune-related dermatitis needs to be monitored with sufficient follow-up and early intervention to prevent the escalation of adverse events. In the occurrence of other adverse events, including liver and kidney function impairment and clinical symptoms such as nausea, vomiting, and diarrhea, the occurrence of the three groups of patients was relatively balanced and controllable.

### 4.2 Comparison of perioperative data related to nICT treatment with nCRT and nCT

Patients enrolled in neoadjuvant therapy are often locally advanced resectable or borderline resectable cases, and one of the main objectives of neoadjuvant therapy is clinical downstaging with the aim of achieving R0 resection; the incidence of R1 resection is roughly between 8 and 20% (20, 21). R0 resection is a key factor that affects the long-term prognosis of patients. A number of studies have shown that R1 resection after neoadjuvant therapy for esophageal cancer is associated with poorer survival, with 5-year OS and DFS of 20–30% and 10–20%, respectively, in patients with R1 resection compared with 40–50% and 30–40%, respectively, in patients with R0 resection (22–24). In this study, two patients underwent R1 resection, which were distributed in the nCRT and nCT treatment groups, and no R1 resection cases occurred in the nICT treatment group. In contrast, in the nCRT treatment group, one patient had an intermediate-to-open thorax surgery because laparoscopy was not feasible.

Another concern for surgeons is whether the surgical challenge increases after neoadjuvant therapy. In this study, the operative time, intraoperative bleeding, and intermediate open/open rates were compared to evaluate the difficulty associated with surgery under the three neoadjuvant treatment modalities. The results showed that the surgical operative time of nCRT was longer than that of nCT, but there was no statistically significant difference compared to nICT, which may be related to the fact that after nCRT, there was an increase in tissue fibrosis, which led to an increase in surgical difficulty. However, there are limited reports on direct comparisons of operative time, intraoperative bleeding, and rates of intermediate open thoracotomy and laparotomy under the three neoadjuvant treatment regimens, and therefore, further studies are needed to investigate the impact of the three neoadjuvant treatment modalities on surgical procedures.

PCR is an evaluation index commonly used in clinical practice to evaluate the efficacy of neoadjuvant therapy for malignant tumors, and it correlates with patients’ EFS and OS (25). After surgical resection in patients with ESCC in the CROSS study, the rate of PCR obtained by patient pathology after nCRT was 49% (8). Similarly, in the NEOCRTEC5010 study, after nCRT in patients with ESCC, the patients’ pathology showed a similar rate of PCR (26). In the same NEOCRTEC5010 study, for ESCC patients after nCRT, a similar PCR rate was obtained for patient pathology, with a result of 43% (12). The nCT has a lower PCR rate of approximately 5–15%, and the clinical downstaging effect is weaker than nCRT (26, 27).

In the present study, the data from the included nCRT study showed a PCR rate at 34.61%; the PCR rate of nICT was 26.92% lower compared to the nCRT treatment, but significantly higher than the 5.13% in the nCT treatment group. The current study showed that nICT has the potential to improve PCR rates (28, 29). As for the comparison of both nCRT and nICT in terms of PCR rate, Wang, et al. (30) and Kong, et al. (31) showed no statistically significant difference between the two in their respective retrospective analysis of data. A meta-analysis showed that the PCR rate after nICT was 31.4% (17). Regarding the reported PCR rate of nCRT treatment, different studies have reported inconsistencies, with the range fluctuating from 29.2 to −43.2% (32), showing a favorable therapeutic effect of nCRT treatment in pathological remission.

According to the preoperative cTNM staging versus pTNM fold plot, nCRT showed a superior downstaging pattern, followed by nICT, whereas nCT had an average downstaging effect. Moreover, nCRT showed better downstaging than nICT, and nICT showed better downstaging than nCT. Further exploration of the comparison of the efficacy of the three treatment modes on T and N staging showed no statistical difference between nCRT and nICT in terms of the downstaging efficacy of T staging. In contrast, both nCRT and nICT showed superior results in T-stage reduction compared to nCT. In N-staging, nICT treatment showed superior results compared with nCRT and nCT. Taken together, this study found that, clinically, nCRT treatment may be chosen for superior efficacy in locally advanced esophageal malignancies with a predominant T-stage, whereas in patients with a predominant N-stage, nICT may have superior outcomes in terms of T-descending efficacy.

Systematic lymph node dissection is the standard treatment for radical esophageal cancer. Abundant lymphatic drainage and the anatomical location of the esophagus may lead to a wide distribution of esophageal cancer lymph node metastases from the neck to the abdomen. This suggests that we need to be vigilant on systematic lymph node dissection, especially under different neoadjuvant treatment regimens and that the distribution of lymph node metastases may differ (33). A study by Evans et al. (34) reported that patients with lymph node regression after neoadjuvant therapy achieved superior survival compared to patients with unresponsive lymph nodes, although the study included patients with adenocarcinoma, which suggests that the lymph node response to neoadjuvant therapy is associated with prognostic relevance. In this study, the number of cleared lymph nodes and the number of groups in the three treatment modalities were lower in the nCRT group than in the other two groups, whereas the number of cleared lymph nodes was higher in patients treated with nICT than in the other two groups. These results may be related to the differences in the mechanisms of action of the respective neoadjuvant therapies. There is no data on whether this difference in the number of cleared nodes leads to a different prognosis.

The incidence of perioperative complications after neoadjuvant therapy for esophageal cancer is a valid indicator of its safety of neoadjuvant therapy. According to the current study data, neoadjuvant therapy did not increase the risk of postoperative mortality compared with surgery alone, but may bring some specific complication risks (35).

Several common clinical perioperative complications were included in the statistical analysis. There were three perioperative deaths with nCRT, one perioperative death with nCT, and no perioperative deaths with nICT. Overall, the perioperative mortality rate with nCRT was relatively high and statistically different from that with nICT. Whether nCRT treatment leads to increased perioperative mortality is controversial. In context, Hamai et al. (36) reported a retrospective analysis comparing nCRT with direct surgical treatment for perioperative complications, and the results of both groups showed no perioperative deaths. Reports have suggested that the use of nCRT should be evaluated with caution and attention to individual patient differences (37).

Among several other major indicators of surgical concern for esophageal cancer, including anastomotic, thoracogastric, and bronchial fistulas, the comparison of the three groups of patients did not show a statistical difference. However, Gronnier et al. (38) reported a comparison of the perioperative complications associated with nCRT versus first-time direct surgical procedures, showing that the incidence of anastomotic fistulas in the postoperative period was 8.8% in the first-time surgical group and 10.6% in the first-time surgical group, and the difference was not statistically significant (*p* = 0.220). However, in cardiovascular complications, celiac chest, and thromboembolism, nCRT was significantly higher than the initial surgical treatment group. No cases of postoperative arrhythmia were observed in the nCRT group, whereas the incidence of postoperative arrhythmia in the nICT group was 12.82%. A study by Gu et al. showed that the incidence of perioperative arrhythmia may be higher with nICT than with other complications (6.3%) (39). In addition, this study found that the incidence of secondary placement of concomitant pleural effusion after nCRT was lower than that in the nICT and nCT treatment groups, which was considered to be related to the mechanism of action of nCRT.

Overall, when comparing perioperative complications among the three neoadjuvant treatment modalities, the perioperative mortality rate may be higher in the nCRT group, and the specific reasons are unknown; however, the rates of several other critical complications did not differ among the three groups of patients. Considering perioperative complications, future studies should focus on patient selection criteria and personalized treatment strategies to improve treatment outcomes and reduce the incidence of adverse events.

### 4.3 Limitations of this study

It is reasonable to speculate that due to the retrospective nature of this study and the small sample size analyzed, the generalizability of the findings is limited. This means that the results may be affected by environmental factors, equipment, and the study personnel. However, retrospective studies pose as foundations for subsequent research and we expect to include a prospective large-scale multicentric study design in the future in order to further validate our findings.

In summary, nICT shows potential in terms of safety, tumor downstaging, and short-term survival. However, its immunity-related toxicity needs to be evaluated. The nCRT remains the “gold standard” for pathological remission, whereas the low PCR rate of nCT highlights the limitations of chemotherapy alone. In clinical practice, the choice of neoadjuvant therapy should be further optimized and comprehensively practiced. Prospective studies with larger sample sizes are needed to verify the long-term benefits of nICT and define the category of patients who will benefit from nICT treatment.

## Data Availability

The raw data supporting the conclusions of this article will be made available by the authors, without undue reservation.

## Footnotes

1
http://ctep.cancer.gov

2
https://cnsknowall.com
